# The role of the bed nucleus of the stria terminalis in the motivational control of instrumental action

**DOI:** 10.3389/fnbeh.2022.968593

**Published:** 2022-11-21

**Authors:** Miao Ge, Bernard W. Balleine

**Affiliations:** Decision Neuroscience Lab, School of Psychology, University of New South Wales, Sydney, NSW, Australia

**Keywords:** bed nucleus of the stria terminalis (BNST), central amygdala (CeA), instrumental conditioning, Pavlovian conditioning, incentive motivation, Pavlovian-instrumental transfer (PIT)

## Abstract

We review recent studies assessing the role of the bed nucleus of the stria terminalis (BNST) in the motivational control of instrumental conditioning. This evidence suggests that the BNST and central nucleus of the amygdala (CeA) form a circuit that modulates the ventral tegmental area (VTA) input to the nucleus accumbens core (NAc core) to control the influence of Pavlovian cues on instrumental performance. In support of these claims, we found that activity in the oval region of BNST was increased by instrumental conditioning, as indexed by phosphorylated ERK activity (Experiment 1), but that this increase was not due to exposure to the instrumental contingency or to the instrumental outcome *per se* (Experiment 2). Instead, BNST activity was most significantly incremented in a test conducted when the instrumental outcome was anticipated but not delivered, suggesting a role for BNST in the motivational effects of anticipated outcomes on instrumental performance. To test this claim, we examined the effect of NMDA-induced cell body lesions of the BNST on general Pavlovian-to-instrumental transfer (Experiment 3). These lesions had no effect on instrumental performance or on conditioned responding during Pavlovian conditioning to either an excitory conditioned stimulus (CS) or a neutral CS (CS_0_) but significantly attenuated the excitatory effect of the Pavlovian CS on instrumental performance. These data are consistent with the claim that the BNST mediates the general excitatory influence of Pavlovian cues on instrumental performance and suggest BNST activity may be central to CeA-BNST modulation of a VTA-NAc core circuit in incentive motivation.

## Introduction

Contemporary analyses of instrumental conditioning suggest that a variety of learning and motivational processes can affect instrumental performance (Balleine, 2019). The focus in recent years has been on the learning processes contributing to the goal-directed and habitual control of such actions, i.e., the relative strength of the response-outcome and stimulus-response associations that support these forms of learning process (Balleine and O’Doherty, 2010; Balleine, 2019). At least as important, however, is the role of various incentive processes, that can modulate performance either through their effects on the *experienced value* of rewarding or reinforcing events directly, or indirectly by modifying the degree to which reward is anticipated or predicted in the environment (Corbit and Balleine, 2016; Balleine, 2019). These latter *predicted values* can exert quite selective effects on action selection through the anticipation of specific events or outcomes. Alternatively, predictions can be more general, being based, not on specific features of rewarding events but on their motivational and emotional effects, something that can alter the state of arousal and so the degree of vigour with which responses are performed (Cartoni et al., 2016; Corbit and Balleine, 2016).

Of these sources of predicted value, there have been several recent reviews of those controlling the influence of identity-specific reward predictions on instrumental performance, focussing mostly on their function in action selection in outcome-specific Pavlovian-instrumental transfer (Holmes et al., 2010; Cartoni et al., 2016; Watson et al., 2018; Balleine, 2019; Eder and Dignath, 2019; Laurent and Balleine, 2021). The current article is instead concerned with the contribution of general incentive processes to performance, i.e., those that induce their effects through a form of energetic shift in motivational or affective arousal. There have been numerous assessments of arousal on instrumental performance over many years of research and any broad attempt to review these issues is beyond the scope of this article (Lang and Davis, 2006; Bradley, 2009; Berridge et al., 2010). It is worth noting here that previous reviews have documented the motiving influence of reward-related contexts and other diffuse predictors (Salamone, 1994; Ikemoto and Panksepp, 1999; Everitt et al., 2003). But, of course, the most thoroughly researched phenomenon demonstrating the influence of affective arousal induced by general reward predictions on instrumental performance comes from assessments of what has come to be called “general” Pavlovian-instrumental transfer (PIT; Dickinson and Dawson, 1987; Dickinson and Balleine, 2002; Corbit and Balleine, 2005, 2011). Here we first provide background to the behavioural assessment of general transfer before consideration of its neural bases.

### General transfer—behavioural factors

The pairing of conditioned stimuli (CSs) with complex multi-faceted unconditioned stimuli (USs) has been demonstrated to produce a similarly complex array of conditioned responses, including those that are US-specific or consummatory in nature, e.g., licking for fluidic outcomes vs. chewing for dry food, and those that are associated with a general appetitive motivational or affective state, which include general search, arousal, and approach responses (Konorski, 1967; Bindra, 1974, 1978; Hearst and Jenkins, 1974; Toates, 1986; Rescorla, 1988; Delamater and Oakeshott, 2007). The consummatory and motivational effects of CSs have been delineated in a number of ways; for example, in the report of distinct signtracking and goal-tracking phenotypes (Jenkins and Moore, 1973; Hearst and Jenkins, 1974; Boakes, 1977), presentation of a localised localized CS, such as light or illuminated lever causes animals variously to approach and contact the CS and to approach the location of impending US delivery. Evidence suggests the former reflects the motivational/emotional and the latter the consummatory influence of the CS: Sign tracking conditioned responses (CRs) are often relatively imprecise or diffuse and less sensitive to changes in US value than goal-tracking CRs (Davey et al., 1989; Chang and Smith, 2016). Other examples have similarly involved manipulations of US “proximity,” either in space or time, with spatial or temporal distance reducing the precision of US-specific CRs and increasing the performance of more general exploratory or activity-related CRs (Konorski, 1967; Vandercar and Schneiderman, 1967; Gast et al., 2016). These kinds of data suggest that Pavlovian CSs can convey distinct forms of information, providing the basis for their differential motivational influence on instrumental performance.

Given this perspective, whereas outcome specific transfer must require sufficiently specific predictions to allow CSs to select actions based on the identity of their consequences, such predictions should not be required for general transfer. Nevertheless, both forms of Pavlovian-to-instrumental transfer evaluate the effects of the interaction between Pavlovian and instrumental conditioning in a test phase in which the effect of the Pavlovian cues on instrumental performance are assessed for the first time (Cartoni et al., 2016). Unlike specific transfer, which typically involves training on two action-outcome associations, general transfer is often demonstrated by examining the excitatory effects of Pavlovian cues on a single action, whether it is trained with the same or a different outcome to that paired with the cue (Estes, 1943; Lovibond, 1983; Hall et al., 2001; Dickinson and Balleine, 2002; Holland and Gallagher, 2003). The Pavlovian phase can establish either different conditioned stimuli paired with distinct outcomes, as has been the case in assessing motivational influences on transfer (Dickinson and Dawson, 1987), or, more frequently, examine the effect of a stimulus paired with an appetitive outcome on instrumental performance against an unpaired control stimulus; one to which the animal has been exposed but not sufficiently for it to become inhibitory (CS_0_). Under these conditions the paired cue typically invigorates the performance of the action relative both to periods without cue presentation and to the unpaired control cue (Cartoni et al., 2016). Importantly, this effect is usually of comparable magnitude regardless of the similarity of the instrumental and Pavlovian outcomes (providing they are similarly valued) and so is usually interpreted as being a product of the appetitive arousal induced by the cue (Rescorla and Solomon, 1967; Dickinson and Balleine, 2002).

This source of appetitive arousal is both in addition to the reward value of the outcome earned by instrumental performance and is gated by primary motivational state. An appetitive cue’s invigoration of single-lever responding can still be observed when the predicted reward is delivered on test (Lovibond, 1983). Furthermore, a cue paired with liquid food or liquid salt when thirsty can elevate performance when animals are subsequently hungry or in a sodium appetite (Dickinson and Nicholas, 1983; Dickinson and Balleine, 1990; Balleine, 1994). Conversely, a cue paired with liquid food when animals are hungry can increase instrumental responding on a pellet-associated lever when tested thirsty (Dickinson and Dawson, 1987). These kinds of data, indicative of what has been called the irrelevant incentive effect (Krieckhaus and Wolf, 1968; Dickinson and Balleine, 2002), demonstrate the motivational control of these forms of general transfer. This degree of control is not observed in specific transfer, which is more strongly regulated by US-specific information than the influence of appetitive motivation. Thus, specific transfer remains largely unaffected by shifts in primary motivation, e.g., from hunger to satiety, whereas this shift can abolish general transfer (Corbit et al., 2007).

### General transfer—neural bases

Despite older claims that general and specific transfer are mediated by a common incentive process, it is clear from the behavioural evidence above and from experiments investigating their neural bases that they are subserved by quite distinct psychological and brain processes. Thus, whereas specific transfer depends on the integrity of basolateral amygdala (BLA; Blundell et al., 2001; Corbit and Balleine, 2005), nucleus accumbens shell (Corbit et al., 2001; Shiflett and Balleine, 2010; Corbit and Balleine, 2011), and their interconnecting pathway (Morse et al., 2020), general transfer has been found to depend on the central nucleus of the amygdala (CeA) and nucleus accumbens core (NAc core; Balleine and Killcross, 1994; Hall et al., 2001; Holland and Gallagher, 2003; Lingawi and Balleine, 2012).

General PIT depends on intact dopamine (DA) transmission: it is abolished by systemic application of D_1_/D_2_ dopamine receptor antagonist flupenthixol in rats (Dickinson et al., 2000; Wassum et al., 2011; Ostlund and Maidment, 2012), and reduced by D_2_/D_3_ receptor antagonist amisulpride in humans (Weber et al., 2016). DA’s role in general transfer is thought to be mediated by NAc core. Bilateral pre-training lesions of NAc core (Hall et al., 2001) or local application of the D_1_ dopamine receptor antagonist SCH-23390 in NAc core on test abolishes general transfer (Lex and Hauber, 2008) whereas the DA agonist amphetamine enhances it (Wyvell and Berridge, 2000). More, direct measurement of DA concentration in NAc core with microdialysis has found that DA level is increased in response to food or drug conditioned cues (Bassareo and Di Chiara, 1999; Ito et al., 2000). Notably, using fast-scan cyclic voltammetry to detect DA release in real time, it has been shown that reward predicting cues induce an increase in phasic dopamine release in NAc core, the amplitude of which positively correlates with lever-pressing rate (Wassum et al., 2013; Aitken et al., 2016). Given the NAc core receives a heavy dopamine innervation from VTA (Beier et al., 2015), mesolimbic DA released into core is considered to underlie the conditioned cue’s general excitatory effect on instrumental actions. Supporting this view, pre-training lesions of VTA reduce general transfer (El-Amamy and Holland, 2007) whereas inactivation of VTA on test abolishes (Murschall and Hauber, 2006) or suppresses it (Corbit et al., 2007).

An important question concerning the neural circuitry underlying general transfer is the brain regions that contribute to the encoding of the cue’s motivational properties. A starting point to address this question is to locate areas that trigger VTA release of DA into NAc core in this effect. Some reports indicate the NAc itself provides one of the heaviest inputs onto VTA DA neurons (Watabe-Uchida et al., 2012), as well as VTA GABA neurons (Xia et al., 2011; Bocklisch et al., 2013; Beier et al., 2015) and so it cannot be ruled out that NAc functions as a controller of DA release into its core division. Apart from NAc core and VTA, a structure that has been repeatedly shown to be indispensable for general transfer is the central nucleus of the amygdala (CeA). Bilateral lesions of the CeA abolish general transfer in rodents (Hall et al., 2001; Holland and Gallagher, 2003; Corbit and Balleine, 2005; Lingawi and Balleine, 2012); and in humans the CeA region is active during a general PIT task in a fMRI study (Prevost et al., 2012). As the CeA lacks direct connections with NAc core (Zahm et al., 1999), it has been proposed that CeA regulates DA release in NAc core through CeA→VTA projections to mediate general transfer (Hall et al., 2001).

The CeA→VTA→NAc core sequential link has also been hypothesised to account for CeA and NAc core’s similar involvement in cue-directed conditioned approach behaviours (Everitt et al., 2000). However, no evidence documenting the functional involvement of this circuit has been published and, indeed, some tracing studies have described CeA’s projection to VTA as light to negligible (Zahm et al., 1999). This picture has, however, been clouded by studies using a rabies strategy to map inputs to VTA showing that CeA sends a moderate input to both VTA DA and GABA neurons, although mostly onto GABAergic neurons (Watabe-Uchida et al., 2012; Beier et al., 2015). Supporting this latter finding is a rather puzzling piece of evidence showing that contralateral lesions of CeA rescued the impairment of general PIT induced by a unilateral VTA lesion whereas an ipsilateral lesion of CeA had no restorative effect (El-Amamy and Holland, 2007). This result suggests that CeA’s direct influence on VTA DA neurons is inhibitory, implying that it interacts with a structure other than the VTA to generate general transfer. In fact, the CeA’s close neighbour within the extended amygdala, the bed nucleus of stria terminalis (BNST), is well positioned to undertake this role.

### The extended amygdala: an anatomical and functional unit

The BNST is a heterogeneous limbic structure that joins the caudal part of the nucleus accumbens shell anteriorly and posteriorly connects with CeA through the fibre tract of the stria terminalis. The parcellation or nomenclature of the BNST is rather inconsistent in the literature. According to the prevailing view, the BNST can be generally divided into medial–lateral and anterior–posterior portions when ontogeny, cytoarchitecture, chemoarchitecture, input, and output connections are taken into considerations (Ju and Swanson, 1989; Ju et al., 1989; Dong et al., 2001a). Because the anterior portion is the area that receives the projection terminals from the CeA, and has been highly implicated in reward processing, our focus is primarily on this area. The anterior BNST can be further subdivided into dorsal and ventral regions based on their positions in relation to the anterior commissure. Anterodorsal (ad), oval (ov), and fusiform (fu) subnuclei within the anterior BNST have received the most attention in recent years following influential studies demonstrating their abilities to shift emotional or motivational state (Tye et al., 2011; Jennings et al., 2013a, b; Kim et al., 2013; Janak and Tye, 2015). As the adBNST and ovBNST make up the majority of the dorsal division, they are often referred to together as dorsal BNST (dBNST). In contrast, the fuBNST is the only nucleus located in the ventral division and is, therefore, referred to as the ventral BNST (vBNST) in most studies. Importantly, both dBNST and vBNST project to the VTA (Silberman and Winder, 2013).

Studies investigating regional or whole BNST’s role in emotional or motivational processes have demonstrated that its functional profile spreads over a wide-range of physiological or pathological behaviours from food intake, mating, arousal, fear, to anxiety (Kalin et al., 2005; Waddell et al., 2006; Davis et al., 2009; Fox et al., 2015), depression-like behaviours (Stout et al., 2000; Hammack et al., 2004), substance abuse disorders (Erb and Stewart, 1999; Aston-Jones and Harris, 2004; Koob, 2008, 2015; Buffalari and See, 2011; Pleil et al., 2015), obsessive-compulsive disorder (van Kuyck et al., 2008; Kohl et al., 2016; Wu et al., 2016; Raymaekers et al., 2017), anorexia (Roman et al., 2012), and pain (Tran et al., 2014). The growing body of evidence on BNST’s functions highlights its potential as a therapeutic target for various maladaptive reward-seeking behaviours and has attracted considerable interest in the mechanism of its regulation over affective or motivational states.

Importantly, in the current context, evidence suggests that the CeA and BNST maintain strong connections; indeed, traditionally, the BNST has been thought of as a downstream output of the CeA (de Olmos and Heimer, 1999) and receives more substantial afferents from CeA than CeA receives from BNST (Oler et al., 2017). Swanson and colleagues view BNST as the pallidal output to CeA’s striatal-like structure (Swanson, 2000; Dong et al., 2001b). In contrast, de Olmos and Heimer (de Olmos and Heimer, 1999) propose that, instead of a simple striatal-pallidal sequential relationship, CeA and BNST maintain multiple symmetrical pairings between sub-nuclei (McDonald, 1983; Holstege et al., 1985; Shammah-Lagnado et al., 2000; Alheid, 2003) with a strong resemblance of cell type and neurochemical makeup within each pair of structures (Alheid and Heimer, 1988; McDonald, 2003) The strong implication is, therefore, that this pair of structures function together as two aspects of a circuit. Considering the less explored status of the BNST relative to the voluminous literature on CeA, this view is particularly helpful in formulating hypotheses with respect to the role of the BNST in emotional or motivated learning. Overall, there is general agreement that the CeA and BNST have similar cortical afferents and subcortical efferents (Gray and Magnusson, 1987; Gray and Magnuson, 1992; McDonald et al., 1999; McDonald, 2003; Nagy and Paré, 2008; Bienkowski and Rinaman, 2013), and strong reciprocal connections (Krettek and Price, 1978; Sun et al., 1991; Sun and Cassell, 1993; Dong et al., 2001b; Dong and Swanson, 2004). Thus, it is safe to assume that BNST should also be functionally linked with CeA, exhibiting a similar functional profile to that of CeA.

In appetitive Pavlovian conditioning, the CeA is involved in assigning conditioned motivation to food predicting cues. CeA lesions impair the acquisition of a visual CS-directed conditioned orienting response, without affecting unconditioned orienting responses to the visual cue (Gallagher et al., 1990). This result has been interpreted as suggesting that the CeA mediates an attentional response to cues (Holland and Gallagher, 1999). The CeA is also involved in the acquisition of conditioned approach responses directed to a localised cue (CS directed sign-tracking CR; Parkinson et al., 2000). Although CeA may not be *necessary* for the expression of a sign-tracking CR, post-training intra-CeA infusion of a dopamine D_3_ receptor agonist enhances CS potentiated food-cup approach behaviours (Hitchcott and Phillips, 1998). In contrast, CeA lesions have no effect over Pavlovian conditioned food-cup approach before the delivery of food (Gallagher et al., 1990), and these US-directed conditioned responses remain sensitive to devaluation (Hatfield et al., 1996). This suggests that the CeA is not involved in the CS’s access to the sensory or incentive value components of the US representation (Cardinal et al., 2002; Everitt et al., 2003). CeA lesions have also been reported to disrupt increments, but not decrements, in conditioned stimulus processing (Holland and Gallagher, 1993a) induced in an unblocking paradigm. Although the processing of a cue is usually blocked when it is presented with a cue that has already been conditioned, if the value of the US is increased or decreased when a second neutral cue is added to the already conditioned CS, processing, and so conditioning, of the second cue is increased. However, in rats with CeA lesions, conditioning of the second cue will only occur when the US value is increased, so called “upshift” unblocking (Holland and Gallagher, 1993a, b). This result suggests that the CeA mediates increases in the associability of the CS (Cardinal et al., 2002). The concept of associability in learning theory denotes a CS’s ability to form associations with the US during conditioning (Pearce and Hall, 1980). In other words, from an error-correction theory perspective of Pavlovian conditioning, the CeA appears to be involved in attributing a positive reward prediction error to the CS.

In contrast to the wide-ranging studies involving CeA, the literature on the BNST’s involvement in appetitive learning is mostly concentrated on its mediation of conditioned place preference (CPP) to natural rewards or drugs of abuse whereas this task has not been the focus of research into CeA function (Jennings et al., 2013a). Nevertheless, CPP is an appetitive contextual conditioning effect (Bardo and Bevins, 2000; Cunningham et al., 2006) supporting the suggested involvement of the BNST in incentive motivation. Nevertheless, the involvement of the BNST in the motivational control of instrumental action and particularly in general transfer effects remains unknown.

### The BNST→VTA pathway

Despite their overall striking similarities, the CeA and BNST maintain dissimilar strengths of connectivity with several key downstream effectors—the paraventricular nucleus of hypothalamus (PVN), substantia nigra pars compacta (SNc), and the VTA. Projections to the PVN from the ventral BNST are particularly massive, whereas few projections from CeA are seen (Gray et al., 1989; Prewitt and Herman, 1998). CeA and BNST have distinct innervation of mid-brain dopamine rich regions like the VTA and SNc. CeM sends considerable efferents to lateral SNc, whereas only few terminalis from BNST end in SNc. In return, the SNc appears to be the only brain region that provides inputs to CeM but not to ventral BNST (Bienkowski and Rinaman, 2013). The CeA’s connections with the SNc are known to be functional and mediate conditioned orienting (Han et al., 1997). Disconnecting CeA from SNc significantly impairs the acquisition of conditioned orienting to auditory cues but preserves food-cup responses (Lee et al., 2005), whereas disconnection of CeA and VTA has no effect on the acquisition of conditioned orienting (El-Amamy and Holland, 2007).

More pertinently, BNST sends prominent projections to VTA. The BNST→VTA pathway has been rigorously demonstrated in rodents in studies utilizing a variety of techniques, including traditional tracing, channel rhodopsin assisted mapping, and a Cre-dependent double-virus strategy (Georges and Aston-Jones, 2002; Dumont and Williams, 2004; Deyama et al., 2007; Jennings et al., 2013a; Kudo et al., 2014; Kaufling et al., 2017; Pina and Cunningham, 2017). Most importantly, manipulations of BNST→VTA pathway potently alter motivational state and reward-seeking behaviours; optogenetic activation of VTA-projecting glutamatergic cells produce real-time place aversion and anxiogenic effects, whereas activation of VTA-projecting GABAergic cells produces place preference and anxiolytic effects (Jennings et al., 2013a). These demonstrations reveal the capacity of BNST→VTA pathway to shift motivational appetitive contextual conditioning. Evidence suggests that CPP largely depends on VTA dopamine transmission. Genetic NMDA receptor knockout on DA neurons dampens burst firing to appetitive cues and induces deficits in CPP (Zweifel et al., 2008). Moreover, direct photo-inhibition of VTA DA neurons supports conditioned place aversion whereas, conversely, phasic activation of VTA DA neurons leads to transient DA release and establishes a place preference in the absence of other rewards (Tsai et al., 2009).

BNST has also been found to mediate the expression of drug CPP, and this effect is likely not induced by BNST’s projection to lateral hypothalamus orexin cells. Instead, disconnection of BNST and VTA impairs the expression of cocaine CPP (Sartor and Aston-Jones, 2012). VTA projecting BNST cells show enhanced c-Fos immunoreactivity during expression of cocaine CPPs (Mahler and Aston-Jones, 2012) whereas inhibition of VTA-projecting BNST cells blocks the expression of CPP to ethanol (Pina and Cunningham, 2017). Adding the fact that BNST can positively regulate VTA DA activity through its dual innervation of VTA GABA and DA neurons, the BNST→VTA pathway appears critical for appetitive contextual conditioning. In addition, the BNST→VTA pathway plays an important role in cue- or stress-induced drug seeking behaviour. Inactivation of BNST attenuates cue- or stress-induced relapse of cocaine-seeking behaviours (Buffalari and See, 2011). VTA-projecting BNST cells show enhanced c-Fos immunoreactivity during cue-induced reinstatement of cocaine seeking (Mahler and Aston-Jones, 2012) whereas disconnection of BNST and VTA reduces stress-induced cocaine seeking (Vranjkovic et al., 2014). Overall, evidence from drug CPP studies suggests that the BNST responds to external and internal cues and regulates drug motivated behaviour through its innervations of VTA (see also Tian et al., 2022).

These various lines of evidence suggest, therefore, that the BNST, the CeA’s close neighbour within the extended amygdala, is a promising candidate structure as a relay of the CeA’s involvement in general transfer. First, the CeA and BNST are tightly interconnected, receiving largely overlapping cortical and amygdala inputs and innervate similar downstream targets, albeit to different degrees. It is, therefore, tempting to speculate that they are involved in similar neurobiological processes. Reports of their roles in appetitive and aversive Pavlovian conditioning provide support for this idea. Second, compared to the CeA, the BNST sends robust projections to VTA, which is a critical locus for general transfer. And optogenetic manipulations of the BNST→VTA pathway potently flip motivational state in real time. Collectively, these studies raise the possibility that the BNST regulates the motivational aspects of general transfer. Given that it remains unclear how CeA interacts with VTA to mediate general transfer, BNST could serve as the missing link for the hypothesised CeA-VTA circuitry. However, whether BNST mediates general transfer has not been assessed.

### The role of the BNST in the motivational control of instrumental performance

Given the claims above, it is tempting to speculate that BNST also regulates the influence of other sources of arousal on the performance of instrumental actions, whether due to Pavlovian cues or *via* other Pavlovian processes embedded in the instrumental conditioning situation (Rescorla and Solomon, 1967). For example, instrumental acquisition can take place in the presence of an explicit discriminative stimulus or an implicit stimulus-outcome relationship between situational stimuli and the reinforcer and in both kinds of situation these stimuli have been found to modulate the vigour of instrumental performance (Bindra, 1978; Colwill and Rescorla, 1988). Furthermore, a context paired with alcohol (Ostlund et al., 2010) or with methamphetamine (Furlong et al., 2017) alters the control of instrumental actions trained in a different context. As discussed previously, evidence suggests that the BNST mediates appetitive contextual conditioning and, therefore, the BNST could theoretically modulate instrumental motivation through its mediatory role in contextual conditioning.

At present there is very little evidence with which to evaluate the role of the BNST in instrumental performance; it is not known whether: (i) instrumental conditioning engages the BNST; (ii) whether any such engagement reflects the conditioned anticipatory or unconditioned features of exposure to the instrumental outcome; and so (iii) whether the BNST is involved in the motivational control of instrumental performance by predictive cues in the general transfer situation.

To address these questions, the current study sought first to examine whether any changes were induced in the activity of neurons in the BNST as a consequence of instrumental conditioning, i.e., as a consequence of mice learning to press a lever for food pellets. We contrasted these changes against those in a yoked control that received matched exposure to reward delivery but for whom lever pressing and rewards were unpaired. There have been reports of robust pERK (phosphorylated extracellular signal-regulated kinase) expression in the dorsal BNST in response to various drugs of abuse (Valjent et al., 2004) and pERK is widely considered as a cellular activity marker for learning and memory (Shiflett and Balleine, 2011a, b). Therefore, pERK was used as the marker of cellular activity for this experiment. The above evidence suggested to us that dorsal BNST was the more likely target of CeA afferents and so of CS-related activity—which turned out to be the case—and so we also used PKC-δ as a marker to delineate both the ovBNST and the lateral region of the CeA within which boundaries pERK+ cells were counted (Wang et al., 2019).

Experiment 2 investigated: (i) the degree to which any changes in activity reflected the amount of instrumental training; and (ii) the anticipation of, vs. exposure to, the instrumental outcome, which we addressed by examining pERK activity in the BNST after a brief period of training, more extended training, and after a brief period of extinction during which the reward was anticipated but no reward exposure was given.

Finally, Experiment 3 examined the functional effects of a lesion of the BNST on Pavlovian conditioning, instrumental conditioning and on the influence of Pavlovian cues on instrumental performance in a general transfer design.

## Materials and Methods

### Animals

Seven to 10-week old male C57B16 mice were acquired from the Australian Research Council (Perth). They were housed in a holding room maintained at 21°C on a 12-h lightdark cycle (lights off at 7 pm). Throughout behavioural experiments the mice were foodrestricted to 85%–90% of their initial weight by giving them 1.5–2.5 *g* of their maintenance chow each day. They were fed after training each day and had *ad libitum* access to tap water while in the home cage. All procedures were approved by the Animal Ethics Committee of UNSW Sydney.

### Apparatus

All behavioural training and testing was conducted in eight identical mouse operant chambers (ENV-307A, Med Associates, Vermont, USA). Chambers were housed in light and sound resistant shells. Each chamber has a house light on one side of the box and a recessed food magazine and two retractable levers on the opposite side with the magazine located in the center and two levers positioning symmetrically on the left and right of the magazine. The reward for all behavioural manipulations was 20 mg grain pellets (Bioserve Biotechnologies, Flemington, NJ, USA), delivered by pellet dispensers into the magazine. The house light and a ventilating fan were turned on throughout all behavioural procedures. Each chamber was also equipped with generators of 3-kHz tone or white noise (~70 dB, Med Associates, Burlington, VT, USA). All chambers were connected to a computer that controlled the equipment and recorded behavioural responses during training using custom codes programmed and run in Med-PC software (Med Associates, Burlington, VT, USA).

### Experimental designs

#### Experiment 1: pERK expression in the BNST and CeA induced by instrumental conditioning

Eighteen mice at 8-weeks of age were evenly assigned to instrumental or yoked training. Mice in the yoked group served as controls for exposure to the various stimulus- and context-reward associations. Each instrumentally trained animal had a corresponding yoked control which had a pellet delivered to the magazine at the same interval as its trained counterpart regardless of whether it pressed the lever or not. All mice were trained for nine daily sessions, including three on continuous reinforcement (CRF), two on random interval (RI)15, one on RI30 and three on RI60. Immediately after the third RI60 session, mice were sacrificed and pERK expression in the BNST and CeA was examined to establish the number of cells displaying pERK immunofluorescence. Sections were also counterstained for PKC-δ as a marker to delineate both the ovBNST and the lateral region of the CeA.

#### Experiment 2: pERK expression at BNST and CeA following extended instrumental training

Eighteen mice were divided to three groups: Trained (*n* = 5), Longer trained (*n* = 5), and Longer trained + test (*n* = 8). Mice in the Trained group underwent an identical instrumental training procedure to Experiment 1. Animals in the other two groups had three more sessions of training on RI60 compared to the Trained group. Animals in Trained and Longer trained groups were immediately sacrificed after the 2nd and 5th RI60 session respectively, whereas the Longer trained + test group were given an additional 5-min extinction test on the day after the 5th RI60 session followed by immediate euthanasia. Again pERK expression in the BNST and CeA was examined in sections counterstained for PKC-δ.

#### Experiment 3: effects of pre-training BNST lesions on general transfer

Surgery was conducted in 20 mice, groups of Lesion (*n* = 12) and Sham (*n* = 8) mice received either bilateral NMDA (10 mg/ml) or vehicle (sterile 0.9% normal saline) injections, respectively, into BNST, 55 nl per side. One week after the surgery, mice were given nine daily instrumental training (3 CRF, 2 RI15, 2 RI30, 2 RI60) sessions before six daily Pavlovian conditioning sessions. Tone and noise were used as the CS and CS_0_ in Pavlovian conditioning. Assignment of auditory stimuli was counterbalanced with lever side and experimental group. On the day after the last Pavlovian session, lever-press performance was tested in extinction in a Pavlovian-instrumental transfer test.

As in a typical PIT paradigm, therefore, the procedure consisted of three phases: instrumental training, Pavlovian conditioning and a transfer test. The procedure adopted a well-established single-lever design (Dickinson et al., 2000; Hall et al., 2001; Holland and Gallagher, 2003) to elicit general transfer, in which performance on one instrumental lever press action was assessed during a CS, a CS_0_ neutral stimulus, and in the absence of both stimuli.

### Instrumental training

Training started with two sessions of magazine training with the outcome delivered on a variable time (VT)-60 schedule during which all mice were familiarised with the chamber environment and learned to retrieve pellets from the magazine. Then they were given 12 daily sessions of instrumental training, in which one lever (left or right) was presented and reinforced with grain pellets. Half of the animals in each group were trained on the left lever and half on the right lever. In the initial 3 days of training, reinforcement was delivered on a CRF schedule, that is, one lever-press lead to the immediate delivery of one pellet. Training sessions ended after 50 pellet deliveries or 60 min, whichever came first. When most mice earned all 50 pellets in a CRF session, they were shifted onto a RI schedule, where the interval between lever-press and reward delivery was random, with an average of 15 s. The training followed a serial progression of increasing interval schedules: three CRF, two RI15, two RI30, and five RI60 sessions. Lever-press and magazine entry events were recorded by the MEDPC program.

### Pavlovian conditioning

After instrumental training, mice went on to receive daily Pavlovian conditioning sessions for a total of 6 days. In each of the first five sessions, there were eight trials of 2-min stimulus (CS), during which pellets were delivered on a random time 30 s schedule. CS trials were spaced with an inter-trial interval (ITI) that averaged 5 min, which included a fixed 2-min period before CS presentation (Pre-CS) serving as baseline. No pellets were given during ITI or baseline periods. On the 6th session, a neutral stimulus (CS_0_) was introduced into the trial sequence. This stimulus was presented twice during the session and so designated as a neutral stimulus or CS_0_. It also lasted for 2 min, but no pellets were delivered. Magazine entries during the stimuli and pre-stimuli periods were recorded.

### Transfer test

Prior to the transfer test, all mice were given one instrumental reminder session where their actions were reinforced with 50 pellets on RI60 as in the last instrumental session before the Pavlovian phase. During the transfer test, their lever-press performance was assessed in extinction with 2-min CS and CS_0_ stimuli presented periodically. The first 9 min of the test was free of stimuli, which was inserted to reduce baseline lever responding. Then four 2-min CS and four 2-min CS_0_ were presented in a pseudorandom order, interlaced with fixed 5-min ITIs, including 2-min Pre-CS or Pre-CS_0_ baseline periods. Stimuli were presented in an S1, S2, S1, S2, S2, S1, S1, S2 order. Lever responding and magazine entries were recorded throughout the session.

### Stereotactic surgery

Mice underwent surgeries at 8–12 weeks of age. They were anaesthetised with 5% isoflurane gas in 100% oxygen (1 L/min) and placed onto the stereotactic frame (Kopf Instruments). Their anaesthetic state was maintained with continuous 0.5%–1.5% isoflurane gas provided by an anaesthetic vaporiser (Ohmeda Tec 5 Anaesthetic Vaporiser Isoflurane). First, the scalp was shaved and disinfected with betadine and 70% ethanol. Then local infiltrative bupivacaine (0.25%, 5 mg/kg) was applied before a small incision was made in the middle of the scalp. Next, a small burr hole was opened with a micromotor drill (Volvere i7), through which a thin glass pipette attached on a nanoliter injector (Nanoject II, Drummond Scientific) was lowered slowly to target coordinates. Last, NMDA was released into targets in 4.6 nl boluses, timed at a rate of approximately 2.3 nl/s. Upon completion of injections, the pipette remained in place for 8 min before removal to minimise track spread. After the surgery, carprofen (1 mg/ml, 5 mg/kg) was given subcutaneously for postoperative analgesia.

Coordinates relative to bregma used for injections were (in mm): anterodorsal BNST (AP +0.14, ML ±1.13, DV −4.20). Coordinates were determined based on a standard mouse brain atlas: The Allen Reference Atlas (Lein et al., 2007) was further adjusted based on the results of pilot surgery. 10 mg/ml NMDA (Sigma-Aldrich, St Louis, MO, USA) was used to create lesions in BNST. NMDA was freshly dissolved in sterile 0.9% normal saline before intracranial injection.

### Tissue processing

Upon completion of the last training session, mice were removed from the chambers and anaesthetised with Lethabarb (300 mg/kg; i.p.). Next, they were transcardially perfused with cold 4% paraformaldehyde (PFA) in 0.1 M phosphate buffer (PB, pH 7.4) for 4 min, brains extracted and post-fixed in the same solution at 4°C overnight. Over the following couple of days, brains were cut into 30-μm coronal sections with Vibratome (VT1000, Leica Microsystems) and stored at −20°C in cryoprotectant (30% ethylene glycol, 30% glycerol, and 0.1 M PB) until they were further processed for immunofluorescence.

### Immunofluorescence

Sufficient sections were taken to cover the ovBNST and CeA regions and were processed to detect of pERK and PKC-δ. Free-floating sections were rinsed in Trisbuffered saline (TBS: 0.25 M Tris, 0.5 M NaCl, 0.1 mM NaF, pH 7.6) three times for 10 min each, followed by 5 min in TBS containing 3% H_2_O_2_ and 10% methanol. After immersed in blocking buffer (0.2% Triton X-100 and 10% normal horse serum in TBS) for 1 h, sections were probed with rabbit anti-phospho-p44/42 MAPK (ERK1/2; 1:1,000; Cell Signaling Technology) and mouse anti-PKC-δ (1:1,000; BD Biosciences) diluted in blocking buffer at 4°C overnight. Next, after three washes in TBS for 10 min each, sections were incubated in blocking buffer containing donkey anti-rabbit Alexa Fluor 546 (1:1,000, Invitrogen), donkey anti-mouse Alexa Fluor 647 IgG (1:1,000, Invitrogen), and Nissl Green (1:1,000, Invitrogen) at 4°C overnight. Then they were washed in TBS for three times, mounted on slides (microscope plain slides, Thermo Scientific) and coverslipped in medium (0.17 mm thickness, Thermo Scientific; Fluoromount-G, SouthernBiotech). For lesion verification in Experiment 3, rabbit anti-GFAP (1:1,000, Sapphire Bioscience) was used as the primary antibody and donkey anti-rabbit Alexa Fluor 488 (1:1,000, Invitrogen) as the secondary antibody. Rest of the procedures were the same as described above.

#### Imaging and cell quantification

Image stacks from both dorsal BNST and CeA were collected from all subjects using a sequential laser scanning confocal microscopy (Olympus FV1000, BX61WI microscope) with 10× (NA 0.40) or 20× objective (NA 0.75). Scan settings of the objective (pinhole size, pixel/μm, laser intensity, and gain) were adjusted following the same procedure for different batches of immunofluorescence and kept the same within the same batch. Donkey anti-rabbit Alexa Fluor 488 and Nissl Green were excited by laser at the wavelength of 473 nm; donkey anti-rabbit Alexa Fluor 546 was excited by 559 nm laser; donkey anti-mouse Alexa Fluor 647 was excited by 635 nm laser. Images in single-slices (10×) or stacks consisting of 2–4 consecutive slices (20×, step size 1.16 μm) were acquired at the dorsal BNST or CeA region respectively. Images taken from both hemispheres of each subject were included for visual inspection and cell quantification. All images were processed and quantified with Open Source Fiji imageJ. Quantification of pERK immunoreactive neurons (pERK+) contained in a stack adhered to the same automatic processing algorithm that projected all cells in a stack onto a 2D image and minimally processed for counting. Size filter was set at 80 μm^2^ for BNST and at 60 μm^2^ for CeA. Results were represented as the number of cells per mm^2^ in the ROI (ovBNST or CeL) within a slice of 1-μm thickness. Numbers of pERK+ neurons in stacks were first averaged within subjects, subject means were then analysed with statistical tests.

#### Statistical analyses

Behavioural and cell count data were analysed in Prism (version 7.0 and 9.0). For comparison of means between two groups, unpaired Student’s t-test, was used. For comparison of means among groups, One-way ANOVA with Brown-Forsythe test of homogeneity of variances or Two-way ANOVA were used, and Tukey test or Sidak’s multiple comparisons test were used for *post-hoc* multiple comparisons. Correlations between independent variables were tested with Pearson’s correlation. *P* < 0.05 was considered as statistically significant in all analyses.

## Results

### Experiment 1

**Figure 1:**
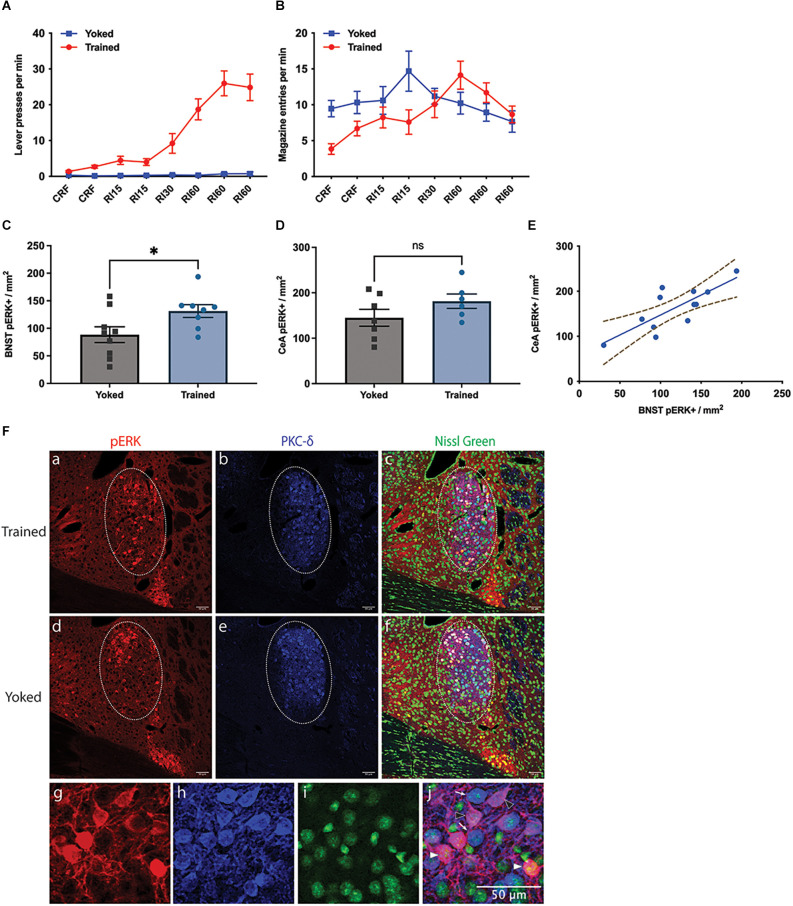
Effect of instrumental training on BNST activity. **(A,B)** Changes in instrumental lever press performance **(A)** and magazine entry **(B)** across the course of instrumental acquisition; **(C–F)** changes in pERK activity in the Oval BNST **(C)** and the CeA **(D)** induced by instrumental training relative to a yoked control; Trained showing significantly higher pERK labelling than Yoked (unpaired t-test, two-tailed, *t* = 2.287, *df* = 15, *P* = 0.037). **(E)** The relationship between pERK activity in the BNST and CeA; significant correlation found between the expression of pERK at ovBNST and at CeL, regardless of training (Pearson’s correlation, *R*^2^ = 0.6118, *P* = 0.0026); **(F)** representative changes in the labelling of pERK (a,d), PKC-d (b,e), Nissl Green (c,f), and colocalisation of pERK and PKC-d (c,f) in the BNST from Trained (top panel) and Yoked (middle panel) animals; PKC-d expression marking the area of ovBNST (b,c,e,f), encircled by dotted line (a–f); (g–j) three subpopulations identified at ovBNST based on the expression of pERK or PKC-d: PKC-d+ /pERK+ (magenta, arrowhead outline), PKC-d+ /pERK- (blue, open arrowhead), and PKC-d-/pERK+ (red, filled arrowhead). All lines and bars presented values as Mean ± SEM. Dotted lines **(E)** represented 95% confidence bands. *P < 0.05. Scale bar: 50 μm, ns: number of subjects in a group.

To search for evidence of the extended amygdala’s involvement in instrumental motivation, we first looked at the expression of pERK in the BNST and CeA following instrumental training and compared the quantity of pERK labelled neurons between trained and yoked groups. The trained group successfully acquired the lever-press action (Figure 1A); lever presses per minute in the final training session was 24.9 ± 3.7 (Mean ± SEM), whereas the Yoked group did not learn the lever action (two-way ANOVA, group and session, group *F*_1,16_ = 40.74, *p* < 0.0001). Both groups exhibited comparable magazine entry rates during the final session (Trained: 8.7 ± 1.2, Yoked: 7.7 ± 1.5 entries/min, Figure 1B).

pERK expression was mostly restricted within ovBNST and ovBNST was clearly demarcated by PKC-δ expression. Only pERK+ neurons within ovBNST were quantified. The trained group had significantly higher pERK expression than the yoked group (Figure 1C), demonstrated in their respective 131.4 ± 11.61 and 88.5 ± 14.31 pERK+ neurons per mm^2^ in ovBNST (unpaired t-test, two-tailed, *t* = 2.287, *df* = 15, *p* = 0.037). As for CeA, mean of pERK+ cells in CeA was 181.5 ± 15.84 in Trained and 144.9 ± 18.61 in Yoked (Figure 1D); however the difference was not significant (unpaired t-test, *t* = 1.467, *df* = 11, *p* = 0.170). Nevertheless, there was a significant correlation between the expression of pERK in the BNST and in the CeA (Figure 1E, Pearson’s correlation, *R*^2^ = 0.6118, *p* = 0.0026). Representative images of pERK, PKC-δ, and Nissl Green staining in the Trained and Yoked groups were shown in Figure 1F. In general, these data demonstrate that pERK activity was increased in the ovBNST by instrumental training and that this increase was over and above that induced by Pavlovian conditioning to any incidental stimuli or to the context or through exposure to the reward alone.

### Experiment 2

**Figure 2:**
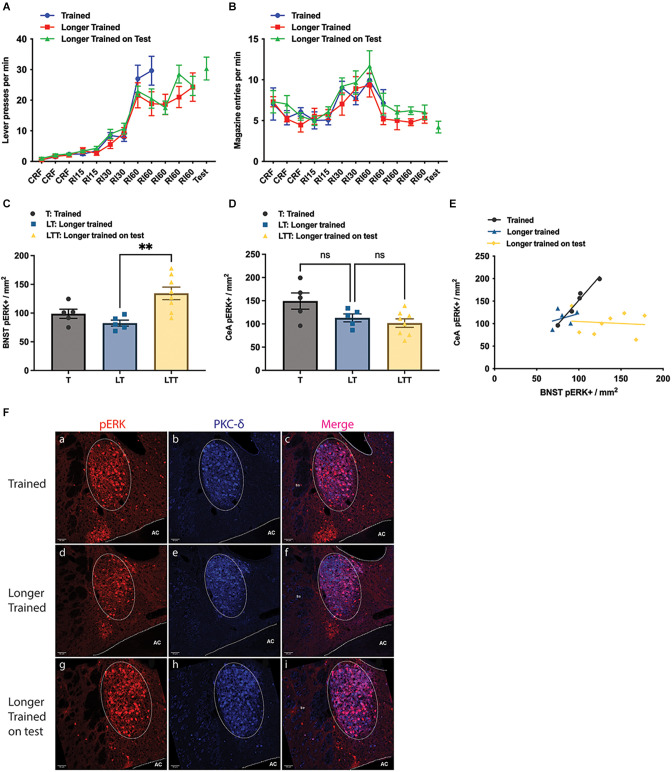
Effect of training and longer training, with and without a brief test in which the outcome was withheld. **(A)** Lever presses per minute across the training phase; **(B)** magazine entries per minute across training; **(C,D)** pERK activity in the Oval BNST **(C)** and in the CeA **(D)** as a consequence of instrumental training (T), longer instrumental training (LT) and longer instrumental training plus an unrewarded test (LTT); LTT exhibiting significantly higher pERK labelling than LT (One-way ANOVA, *F* = 8.041, *P* = 0.0042; *post-hoc* Tukey’s test, LT vs. LTT difference −51.94, *P* = 0.0046). **(E)** The relationship between pERK activity in the BNST and CeA; significant correlation was only found in the Trained (Pearson’s correlation, *R*^2^ = 0.9653, *P* = 0.0028); **(F)** representative images taken from group T, LT, and LTT (top, middle, and bottom panel respectively) showing labelling of pERK (a,d,g), PKC-d (b,e,h), and colocalisation of pERK and PKC-d (c,f,i); PKC-d expression (b,c,e,f) marking the area of ovBNST, encircled by dotted line (a–f). All lines and bars presented values as Mean ± SEM. AC, anterior commissure. **P < 0.01. Scale bar: 50 μm, ns: number of subjects in a group.

To examine how BNST’s activity changes with extended instrumental training, under rewarded vs. unrewarded conditions, we next compared pERK expression in BNST and CeA in mice given instrumental training (group Trained = group T), extended instrumental training (group Longer-Trained = group LT), and those with extended training plus a brief additional test during which the outcome was withheld (group Longer Trained on Test = group LTT). All three groups successfully learnt the lever-press action (Figure 2A). Press rate was transiently lower in LTT and LT vs. T groups on the 2nd RI60 session (two-way ANOVA, group and session, interaction *F*_16,120_ = 1.789, *p* = 0.0401), however all three groups showed comparable press rates on their final session of training: 29.7 ± 4.7 in T; 24.3 ± 4.6 in LT and 30.4 ± 3.7 in LTT (one-way ANOVA, *F* < 1). The groups showed similar rates of magazine entry across acquisition (Figure 2B).

Quantification suggested that the number of pERK+ neurons in the three groups differed.

The average pERK+ neurons in ovBNST was 98.8 ± 8.05 in group T, 82.4 ± 5.25 in group LT, and 134.4 ± 10.95 in group LTT (Figure 2C). The LTT group showed significantly higher pERK expression than the other two groups (one way ANOVA, *F* = 8.041, *p* = 0.0042; Tukey’s test: LT vs. LTT difference = −51.94, *p* = 0.0045; T vs. LTT difference = −35.58, *p* = 0.0486). On the other hand, the number of pERK+ neurons in the CeL was not significantly different between the group T and LT, or between the group LT and LTT (one-way ANOVA, *F* = 4.338, *p* = 0.0326; Tukey’s multiple comparisons, T vs. LTT, difference = 47.56, *p* = 0.0275), with a mean number of 149.3 ± 17.57 in the group T, 113.0 ± 8.58 in the group LT and 101.7 ± 9.16 in the group LTT (Figure 2D). Additionally, as in Experiment 1 we found a significant positive linear relationship between the number of pERK+ neurons in the CeA and pERK+ neurons in the ovBNST in Group T (Pearson’s test, *R*^2^ = 0.9653, *P* = 0.0028) but not in either Group LT or LTT, suggesting that any relationship between CeA and BNST declines with overtraining (Figure 2E). Representative images of pERK, PKC-δ staining in the ovBNST for each of the groups in Experiment 2 are shown in Figure 2F. Generally, these data confirm that BNST was highly activated during instrumental performance but that this activity was greater during a test in which the outcome was anticipated but not delivered. This is consistent with the argument that the BNST is activated by the influence of incentive processes associated with the prediction of reward on instrumental performance.

### Experiment 3

#### Lesion assessment

**Figure 3:**
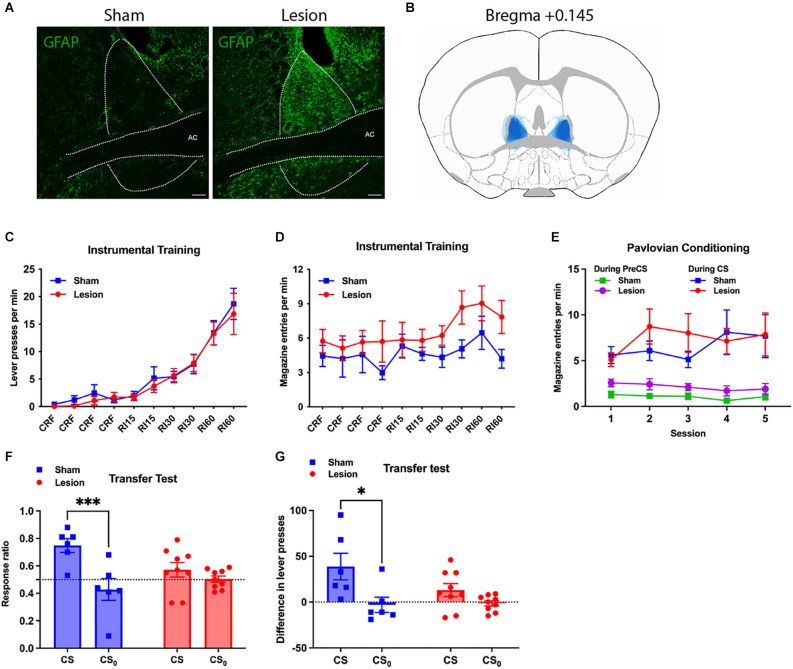
Effect of NMDA-induced lesion of BNST on general Pavlovian-instrumental transfer. **(A)** Representative images of the BNST showing GFAP activity in the Lesion and Yoked groups; **(B)** reconstruction of the lesion in BNST by overlapping lesion placement of all subjects in Lesion group (in blue); **(C,D)** performance during the instrumental training phase showing lever presses per minute **(C)** and magazine entries per minute **(D)** across sessions; **(E)** conditioned magazine entry responses performed during the Pavlovian conditioning sessions in the Lesion and Sham Groups showing pre-CS baseline performance and performance during the CS; **(F)** responding during the test of general Pavlovian-instrumental transfer showing the effects of BNST lesions on the elevation in response vigour during the CS and CS_0_ relative to baseline using an elevation ratio: (responding during the CS)/(responding during CS+ responding during the baseline); transfer effect found impaired in Lesion (Two way ANOVA, CS × Lesion interaction, *F*_1,13_ = 7.868, *P* = 0.0149); **(G)** shows the same data during the transfer test except using a straight subtraction of CS—baseline responding; transfer effect observed only among the Sham (Sidak’s multiple comparison test, *t* = 2.960, *df* = 13, adjusted *P* = 0.0220). All lines and bars presented values as Mean ± SEM. ^*^P < 0.05, ***P < 0.001. AC, anterior commissure. Scale bar: 100 μm.

Representative images from the lesioned and sham groups and reconstruction of BNST lesions in the lesioned group are shown in Figures 3A,B. BNST lesions were confirmed by visual inspection using GFAP immunofluorescence on three coronal sections (bregma +0.245, +0.145, +0.020) in each subject. Three subjects from the Lesion and one from the Sham group were excluded from behavioural analyses due to either faint GFAP signals or to major spread of the signal into the striatum meaning nine and six mice remained in Lesion and Sham groups, respectively.

#### Behavioural results

Figures 3C–E present the data from the instrumental training and Pavlovian conditioning phases of this experiment. Both BNST Lesion and Sham groups showed rapid acquisition of lever-pressing over instrumental sessions (Two-way ANOVA, lesion and session, session *F*_9,117_ = 31.83, *p* < 0.0001, lesion *F*_1,13_ = 0.2054). Press rate on final RI60 training session was 16.9 ± 3.8 (Mean ± SEM) presses/min and 18.7 ± 2.8 presses/min in Lesion and Sham groups, respectively (Figure 3C). Corresponding magazine entry rates during the final session were 7.8 ± 1.4 and 4.2 ± 0.8 entries/min. Although entry rates appeared to be higher in the lesion vs. Sham group across sessions, this difference was not significant (Figure 3D, Two-way ANOVA, lesion and session, lesion *F*_1,13_ = 3.502, *p* = 0.084).

Again, during Pavlovian conditioning the Lesion Group showed a slightly higher entry rate during the Pre-CS period compared to the Sham Group (Two-way ANOVA, lesion and session, lesion *F*_1,13_ = 5.076, *p* = 0.0422), with an average of 1.89 ± 0.6 and 1.06 ± 0.35 entries/min (Figure 3E). During CS presentation, however, both groups entered the magazine at a similar rate across sessions (two-way ANOVA, lesion and session, lesion *F* < 1), with 7.9 ± 2.3 entries/min in Lesion and 7.7 ± 2.3 entries/min in Sham. Entry rate was significantly higher during the CS than the Pre-CS period, in both the Lesion and Sham groups (two-way ANOVA, session and CS presentation in Lesion, CS presentation *F*_1,16_ = 12.18, *p* = 0.0030; two-way ANOVA, session and CS presentation in Sham, CS presentation *F*_1,10_ = 16.85, *p* = 0.0021). As such, despite the slight increase in baseline magazine entries in the lesion group, there was no evidence that Pavlovian conditioned responding differed in the two groups.

Results of the PIT test are plotted in Figures 3F,G. During this test, the Lesion group had a lever-press rate of 5.3 ± 0.9 presses/min during CS and 3.6 ± 0.4 during the Pre-CS baseline, relative to Sham’s 6.9 ± 2.0 during CS and 2.0 ± 0.5 during Pre-CS. Both groups had comparable lever-press rate during CS_0_ (Lesion 3.6 ± 0.6, Sham 3.8 ± 1.2) or Pre-CS_0_ (Lesion 3.8 ± 0.8, Sham 4.2 ± 0.8). Transfer was measured as the ratio of lever-presses during CSs to total lever presses during the CSs plus the preceding Pre-CS period (Figure 3F). The Lesion Group had response ratios of 0.57 ± 0.05 during CS and 0.50 ± 0.02 during CS_0_, whereas the Sham group had ratios of 0.75 ± 0.05 during CS and 0.43 ± 0.08 during CS_0_. Two-way ANOVA found that the CS, relative to CS_0_, significantly elevated the response ratio (stimulus and lesion as two factors, stimulus *F*_1,13_ = 18.87, *p* = 0.0008), demonstrating successful generation of general PIT with the current experimental procedure. Importantly, a significant interaction between stimulus and lesion was found (*F*_1,13_ = 7.868, *p* = 0.0149). Furthermore, whereas the response ratio during CS did not differ from that during CS_0_ in the Lesion Group (Sidak’s multiple comparison test, *t* = 1.217, *df* = 13, adjusted *p* = 0.4305), it was significantly increased from that during CS_0_ in the Sham Group (*t* = 4.615, *df* = 13, adjusted *p* = 0.001), suggesting transfer was impaired in Lesion while preserved in Sham. Comparable results were found when we subtracted the pre-CS baseline from responding during the CS and CS_0_ (Figure 3G). Lever presses were increased during CS compared to CS_0_ (two-way ANOVA, stimulus and lesion, stimulus: *F*_1,13_ = 9.517, *p* = 0.0087). A transfer effect was observed in the Sham Group (Sidak’s multiple comparison test, *t* = 2.960, *df* = 13, adjusted *p* = 0.0220) but not the Lesion Group (*t* = 1.252, df = 13, *p* = 0.411).

This experiment assessed the functional effects of dBNST lesions on general Pavlovian-instrumental transfer. Although no effects of the lesion were found on baseline instrumental performance or on the influence of CS_0_ on that performance, lesions of dBNST significantly reduced the excitatory effect of a CS on that performance and so significantly attenuated the general transfer effect. As anticipated by our presentation of the literature above, therefore, these results suggest that the BNST mediates the influence of incentive motivation on instrumental performance.

## Discussion

This series of studies was developed based on a review of the literature on the function of the BNST in incentive motivation. Current evidence suggests that the BNST plays a significant role in the way Pavlovian cues alter the vigour of instrumental actions. To assess this we examined three questions: (i) what impact does instrumental training vs. yoked exposure to the instrumental outcome have on activity in the BNST? (ii) are any changes in BNST activity increased by longer training or are they merely related to the degree of outcome anticipation? and (iii) is the influence of Pavlovian cues on instrumental performance sensitive to lesion-induced damage to the BNST?

Experiment 1 found increased pERK+ cells in the ovBNST in instrumentally trained compared to yoked controls. A straightforward interpretation of this finding is that this ovBNST activity reflects added processes in the trained relative to the yoked condition. Trained mice differed from yoked mice in processes related to instrumental learning, which includes but is not limited to initiation and execution of the action, and evaluation of the outcome. Given the BNST’s broad involvement in motivated behaviour and our previous conclusion regarding the role of the BNST in the influence of conditioned motivation on instrumental actions, this result suggests that ovBNST’s activity likely indicates the motivational control of instrumental action. A number of studies link the dorsal division of BNST to the modulation of instrumental vigour. For example, Dumont et al. (2005) found an elevated NMDAR/AMPAR ratio in dorsal BNST following instrumental learning for cocaine reward. Importantly, the NMDAR/AMPAR ratio, which reflects neuroplasticity, positively correlated with instrumental vigour for cocaine reward. This report suggests that dorsal BNST could be an important locus that psychostimulants modify to generate heightened or sensitised responding. Also, because pERK expression follows activation of NMDARs, as seen in striatum, increased pERK expression in ovBNST among instrumentally trained animals, as observed in our experiment, was likely a product of a similar process of NMDAR upregulation. It is worth noting, however, that Dumont et al. (2005) failed to observe a change in NMDAR/AMPAR ratio in subjects who were trained to press for a natural reward of sucrose. There are few reports of BNST’s involvement in instrumental conditioning, which is in stark contrast to the bulk of the literature which focusses on its role in the effects of stress or drugs of abuse on various reward-seeking behaviours. This discrepancy raises the possibility that the BNST is especially vulnerable to influences from neuromodulators or psychoactive agents. Overall, ovBNST’s activity in instrumental learning, as indexed by increased pERK expression, can be reasonably interpreted as evidence of BNST’s role in instrumental motivation.

Next, in Experiment 2, ovBNST showed a higher degree of pERK activity after instrumental performance had been tested in the training context when reward was anticipated but withheld (Group LTT) than when reward was actually delivered during training (Group LT). The final press rates in the T vs. LT vs. LTT Groups did not differ significantly in this experiment, suggesting that the increased proportion of pERK in ovBNST with reward withheld had little to do with instrumental vigour. Furthermore, pERK expression was, if anything, slightly reduced in mice in the LT Group (i.e., 5× RI60 sessions vs. 2× RI60 sessions) and so changes induced by training itself or extended access to reward appear to have had little impact in themselves. Instead, and particularly given the brevity of the extinction test, it seems likely that it was the prediction of reward *in the absence of its delivery* that provoked the considerable increase in pERK activity in the LTT Group. Nevertheless, it is unclear precisely what role the absence of reward played in this finding: i.e., whether withholding reward enhanced its anticipation or increased the saliency of reward predictors by increasing ambiguity or uncertainty, something that has recently been linked to BNST in aversive situations (Figel et al., 2019; Goode et al., 2019; Naaz et al., 2019).

Given this finding and from the perspective of our analysis of the literature on the extended amygdala, particularly BNST’s highly interconnected and mirrored relationship with CeA, we hypothesised that BNST plays a similar role as CeA in general transfer. It is well established that pre-training lesions of CeA abolish general transfer. Therefore, pre-training lesions of BNST were predicted to disrupt general transfer and, indeed, we found just this effect. Although the lesion was aimed at dorsal BNST, and the majority of the damage was localised there, there was some invasion of ventral BNST and so the precise source of the effect remains unclear. Nevertheless, this result adds weight to the view that the two structures are functionally linked and increases the likelihood that BNST relays CeA’s influence on general transfer. Indeed, CeA participates in the encoding of the CS’s motivational properties and is essential for the acquisition of CS-directed conditioned approach (sign-tracking CR; Cardinal et al., 2002). Furthermore, as discussed above, the motivational properties attributed to the CS are likely to constitute the invigorating power supporting general transfer and, if CeA lesions undermine general transfer by preventing the establishment of this CS-elicited motivation, then our result suggests that the effects of BNST lesions may have also been mediated by the CS’s acquisition of motivational properties.

Aside from the CeA, the NAc core has been recognised as a key correlate for the expression of general transfer. In the same manner as CeA and NAc core (Hall et al., 2001), we found that pre-training lesions of BNST did not significantly affect Pavlovian conditioning or instrumental acquisition but attenuated general transfer. The BNST’s remarkable similarity to the CeA and NAc core in terms of selective involvement in general transfer encourages the view that the BNST belongs to a functional circuit that includes CeA and NAc core to modulate the general transfer effect. Importantly, there is no evidence in any of these studies to conclude that the BNSTs effects or those of any of its affiliated structures are involved in Pavlovian conditioning *per se*. Rather it appears that this circuit mediates a specific aspect of appetitive motivation; the arousal generated by Pavlovian predictors. Thus, conditioned responding during Pavlovian conditioning was unaffected by BNST lesions whereas, in contrast, the influence of that conditioning on instrumental performance was strongly attenuated.

On the other hand, in instrumental training, the press rate of subjects with BNST lesions was numerically—if not significantly—lower than that of sham controls, as has been previously reported with NAc core lesions (Hall et al., 2001). It has been proposed that the minor reduction in instrumental responding seen in animals with NAc core lesions results from impaired context conditioning (Balleine and Killcross, 1994; Aberman and Salamone, 1999). Since there is considerable evidence showing the BNST plays an important role in appetitive context conditioning, it is likely that BNST lesions mildly affect instrumental vigour in the same way and for the same reason as those of the NAc core.

Our result positions BNST in the encoding of CS’s motivational properties, and such a role is likely to be amplified by BNST’s descending connections with VTA. BNST both sends and receives robust projections to and from VTA GABA and DA neurons, which enable BNST to exert a direct influence over DA release (Melchior et al., 2021; Yu et al., 2021). VTA-projecting BNST neurons are overwhelmingly GABAergic and these neurons preferentially synapse onto VTA GABA neurons. About 70% of VTA GABA neurons are responsive to stimulation of GABAergic terminals from BNST and optogenetic stimulation of BNST GABAergic inputs to the VTA is rewarding and anxiolytic, effects similar to those resulting from optogenetic inhibition of VTA GABA neurons (Jennings et al., 2013a). Therefore, activation of BNST projection neurons to VTA likely disinhibits VTA DA neurons leading to increased DA activity in its targets including NAc core.

## Implications

Our results provide the first evidence to our knowledge of BNST’s contribution to general transfer and encourage positioning BNST within the theoretical circuit mediating transfer. In particular, the results are in line with our argument that the CeA mediates general transfer through its connections with BNST, implying a place for the extended amygdala in the acquisition of the motivational properties of a conditioned stimulus. Future research is needed to flesh out the BNST’s role in general transfer and shed light on the neural mechanisms underlying its influence. As BNST takes part in a wide array of motivated behaviours, understanding its role in the neural bases of conditioned motivation will have broad implications in elucidating the pathogenesis of the dysfunctional responding commonly seen in psychological disorders, and will be fruitful in developing strategies to restore normal motivational control.

For example, as noted previously, general transfer is thought to underlie maladaptive behavioural responding in various psychiatric conditions, such as stress and anxiety (Pool et al., 2015; Quail et al., 2017), drug addiction (Belin et al., 2009; Hogarth et al., 2013; Ostlund et al., 2014), alcohol use disorder (Corbit and Janak, 2007; Garbusow et al., 2016), and bipolar disorder (Hallquist et al., 2018). Given our conclusion that BNST mediates general transfer and possibly regulates instrumental motivation, there should be evidence indicating that the BNST plays a role in these same conditions. And, indeed, there are reports that the BNST plays a crucial role not only in the regulation of anxiety (Tye et al., 2011; Yassa et al., 2012; Grupe and Nitschke, 2013; Jennings et al., 2013a; Kim et al., 2013), drug-seeking behaviours (Avery et al., 2016; Daniel and Rainnie, 2016; Gungor and Pare, 2016; Mantsch et al., 2016), but also in binge-drinking (Pleil et al., 2015; Rinker et al., 2017), binge-eating (Jennings et al., 2013b; Micioni Di Bonaventura et al., 2014), anorexia (Sweeney and Yang, 2015), excessive water drinking-related compulsive behaviours (van Kuyck et al., 2008; Wu et al., 2016), and OCD (Kohl et al., 2016; Luyten et al., 2016; Raymaekers et al., 2017). Many of these conditions arguably share a basis in maladaptive instrumental responding. A deeper understanding of the BNST’s role in instrumental processes is therefore of the highest importance and may prove fruitful in elucidating the pathological mechanisms underlying these conditions.

## Data Availability Statement

The raw data supporting the conclusions of this article will be made available by the authors, without undue reservation.

## Ethics Statement

The animal study was reviewed and approved by UNSW Sydney Animal Ethics Committee.

## Author Contributions

MG conducted the research and wrote the manuscript. BB wrote the manuscript and supervised the research project. All authors contributed to the article and approved the submitted version.

## Funding

This research was supported by grants from the Australian Research Council, #DP160105070 and #DP200103401, and by a Senior Investigator Award from the National Health and Medical Research Council of Australia to BB, #GNT1079561.

## Conflict of Interest

The authors declare that the research was conducted in the absence of any commercial or financial relationships that could be construed as a potential conflict of interest.

## Publisher’s Note

All claims expressed in this article are solely those of the authors and do not necessarily represent those of their affiliated organizations, or those of the publisher, the editors and the reviewers. Any product that may be evaluated in this article, or claim that may be made by its manufacturer, is not guaranteed or endorsed by the publisher.
